# The Management of Catatonia in Bipolar Disorder with Stimulants

**DOI:** 10.1155/2015/423025

**Published:** 2015-02-19

**Authors:** Waheed K. Bajwa, Ali Rastegarpour, Omar A. Bajwa, Jessica Babbitt

**Affiliations:** Cary Behavioral Health P.C., 160 NE Maynard Road, Suite 200, Cary, NC 27513, USA

## Abstract

Catatonia, while not a rare occurrence in bipolar disorder, has not been widely discussed in the literature. We present a case of a married Caucasian male with a history of bipolar disorder, exhibiting catatonia and experiencing difficulty in day-to-day functioning. He demonstrated impairment in cognition and an inability to organize simple activities of daily life. After exhausting a number of options for medical management, including benzodiazepines, atypical antipsychotics, and amantadine, he only displayed significant clinical improvement with the addition of a stimulant, methylphenidate. In time, the patient saw a complete return to normal functioning. The use of stimulants for catatonia in bipolar disorder may be an interesting and effective option for treatment. While this is not the first time this treatment has been suggested, there is very little data in support of it; our case confirms the discoveries of previous case reports.

## 1. Introduction

The management of catatonia in bipolar disorder lacks a clear consensus, especially as more popular options are gradually exhausted. In terms of prevalence, catatonia has been reported to be present in as many as 28% of all patients with bipolar disorder [1]. In a report from 2003, symptoms of catatonia, grossly disorganized behavior, and negative symptoms occurred in 21% of patients with psychosis. Catatonia was reported with higher prevalence in bipolar patients with a history of psychosis, which may in turn affect more than half of all patients with bipolar disorder [2]. In addition, catatonia has been associated with both manic and depressive states of bipolar disorder [3]. In one study of actively manic bipolar patients, 19 of 61 patients were diagnosed with catatonic mania [4]. These catatonic manic patients had more mixed episodes, more severe manic symptoms, more general psychopathology, higher prevalence of comorbidity, longer hospitalization, and lower Global Assessment of Functioning (GAF) scores than their noncatatonic counterparts.

## 2. Case Presentation

Since March 2014, a 55-year-old, married, Caucasian male has been followed up at our outpatient psychiatry practice after discharge from inpatient treatment. He was a tenured faculty member at a well-respected university with research and teaching responsibilities. He led a socially active lifestyle and liked to play sports.

The patient developed his first episode of mania with psychosis in 2012. He received a trial of risperidone, but it was discontinued after he developed severe extrapyramidal symptoms (EPS). He responded well to lithium and was maintained on it for a year, after which, the patient discontinued its use because he questioned his diagnosis and the need for ongoing treatment. The patient relapsed in February 2014. His symptoms included a subjective increase in energy level, hypersexuality, religious preoccupation, grandiosity, and argumentative behavior. He complained of having too many noisy thoughts in his head, talked about a “logical paradox,” and was constantly distracted. He was sleep-deprived during a lecture tour and attempted to self-medicate by taking a larger quantity of lorazepam than he was prescribed. His socially inappropriate behavior, including use of inappropriate language and sometimes awkward undressing, was concerning to his family. The patient was hospitalized at a local hospital and, upon completion of inpatient treatment, released to outpatient follow-up at our practice.

The patient had a prior history of mania and psychosis and he ultimately became unable to function at work. He was experiencing difficulty in performing activities of daily living (ADLs) and could not complete simple tasks such as doing the laundry, making a sandwich, or driving to and from the grocery store. He displayed catatonia, poverty of speech, and a sense of fear and embarrassment in public. Mental status examination showed classic features of catatonia, characterized by stupor, mutism, negativism, mannerism, and grimacing. There was no report of auditory or visual hallucinations, but there was significant impairment in cognition demonstrated on the Montreal Cognitive Assessment (MoCA) test, revealing a score of 20/30 with difficulties in serial sevens, language, attention, visuospatial, and executive functions. Apart from the items described, he had no relevant psychiatric history, including no history of attention deficit disorder or substance abuse or dependence.

The patient was referred for neurological evaluation. His neurological workup was normal and his MRI did not reveal any abnormalities. The patient was initially maintained on lithium (at a serum level of 0.5 mEq/L) and given lorazepam to address the catatonia. The patient's wife always accompanied him to appointments and would describe the patient's symptoms for him. His presentation was generally noncommunicative. During the initial sessions, he lacked spontaneity; he did not respond to direct questioning most of the time. He spoke in a low monotone voice, primarily to express his concerns about medications. He was unable to explain his inability to perform simple tasks. The medications had no effect on his catatonia. Ziprasidone (Geodon) was added for the patient but was discontinued soon after due to side effects of nausea, stiffness, and tremors. Subsequently, asenapine (Saphris) was initiated.

The patient was later given a trial of vitamin E and amantadine. He experienced minor improvement in organization and social interactions but maintained a presentation of catatonia, lack of spontaneity, impaired organization, and the inability to plan tasks. The patient appeared to deteriorate after briefly discontinuing the asenapine. He appeared internally preoccupied, his catatonia had intensified, and bradykinesia was prominent.

The patient was placed in an intensive outpatient program and restarted asenapine. He had discontinued amantadine due to personally feeling that he was taking too many medications. Armodafinil (Nuvigil) was consequently added to address the patient's catatonia. The patient did not have any improvement and appeared to be more anxious. He could not function independently and could not drive. He felt dull, lacked motivation, and continued to fear being embarrassed in public settings. At this point, the patient stopped taking lithium due to his worries of potential side effects. He complained of a metallic taste and tremors and expressed concern about kidney function and thyroid suppression.

While maintaining asenapine at 5 mg, methylphenidate was added to the patient's regimen at a dose of 10 mg daily. The improvement with methylphenidate was dramatic. The patient's catatonia was resolved and there was significant improvement in cognition, organization, motivation, and motor activity. The patient was followed up after two weeks. He had started driving again and had begun to initiate other activities in his life. He returned to his office and started responding to his e-mails and had also returned to recreational sports. He initiated a request to return to work. The patient started to make jokes again. After returning to work on a full-time basis, methylphenidate was gradually tapered, and after achieving a therapeutic dose of lamotrigine, the asenapine was additionally tapered off.

The patient returned to full functioning and was placed on lamotrigine (50 mg daily) for maintenance therapy. During his most recent visit he was alert, pleasant, and spontaneous with normal psychomotor activity, fluent speech, and full range of affect without evidence of internal preoccupation.

## 3. Discussion

The criteria for a diagnosis of catatonia were recently changed in the DSM 5. The symptom sets were removed and catatonia was defined as the presence of three or more of 12 symptoms including catalepsy, waxy flexibility, stupor, agitation, mutism, negativism, posturing, mannerisms, stereotypies, grimacing, echolalia, and echopraxia. This change was primarily designed to address the underrecognition of catatonia by simplifying and hence adding to the practicality of diagnosing catatonia, both as an independent entity and as a specifier of concurrent disorders [5].

There is no single recommended guideline for the treatment of catatonia occurring in bipolar disorder. The first line of treatment typically consists of a benzodiazepine [6, 7]. For these means, both lorazepam and diazepam have been demonstrated to be effective and their consecutive use has been proposed as a first step in the management of catatonia in bipolar disorder [8]. In our patient, lorazepam did not prove effective as a first line treatment.

Patients may also be placed on mood stabilizers for the control of the underlying bipolar disorder, but there is not considerable evidence to show that mood stabilizers have any effect in treating the catatonia in bipolar disorder [9]. This was the case in our patient, who did not show any signs of improvement on lithium.

Many studies have reported an improvement of catatonia in bipolar disorder with atypical antipsychotics. Ziprasidone [7] and quetiapine [10] have both been used with varying success for catatonia in bipolar disorder. Interestingly, conflicting studies have shown that catatonia in bipolar disorder may be worsened by antipsychotic medications [3]. This can be problematic because catatonia is frequently misattributed to schizophrenia as opposed to bipolar disorder. One study found that, of twelve patients with episodes of catatonia, who were admitted to inpatient units, eight were initially diagnosed as schizophrenic. Within the course of two years, these same patients were ultimately diagnosed with bipolar affective disorder [11]. It should be noted that atypical antipsychotics were also not effective in our patient.

Amantadine, which was also tried for our patient without any success, is another medication that has been suggested to be effective for catatonia in bipolar disorder in the past [12].

Ultimately, the patient's catatonia responded to a stimulant: methylphenidate. To our knowledge, the treatment of catatonia in mood disorders using stimulants has been reported twice before in previous literature. In 2010, Prowler et al. [13] reported a case of depression with catatonia in an elderly patient. The patient was nonresponsive to lorazepam but experienced a marked and rapid response to methylphenidate. In 2012, Neuhut et al. [14] reported a case that replicated these results in a patient with bipolar disorder. This patient was nonresponsive to benzodiazepines and was unable to receive electroconvulsive therapy (ECT). He was given methylphenidate 5 mg and, similar to the case presented by Prowler et al., experienced a rapid response.

Neuhut et al. [14] propose that the presumed pathophysiology for catatonia development is one of three hypothesized mechanisms. The first hypothesis includes decreased GABA-A in the right lateral orbitofrontal and parietal cortex. This hypothesis is supported by the efficacy of the two widely recognized evidence-based managements, benzodiazepines and ECT. The second is increased glutamate in the striatum, which provides a basis for the efficacy of glutamate antagonists such as amantadine, topiramate, and gabapentin. The third hypothesis provides the rationale for stimulant treatment in catatonia and consists of decreased dopamine activity in the mesostriatum. The “diminished dopamine hypothesis” explains a mechanism by which dopamine agonists, such as carbidopa, bromocriptine, selegiline, and amantadine, can be effective, while D_2_ blockers, such as haloperidol, may exacerbate the catatonia.

This is the third reported case of the use of stimulants for catatonia occurring in mood disorders and only the second that documents its use in bipolar disorder. We think that replicating these results is an important finding that necessitates further studies and larger trials to determine its efficacy. If proven, the use of stimulants to combat catatonia in bipolar disorder could have tremendous implications and obviate the need for ECT in many patients.
